# Supplementary material for: An LH1–RC photocomplex from an extremophilic phototroph provides insight into origins of two photosynthesis proteins
Source: Commun Biol. 2022 Nov 7;5:1197. doi: 10.1038/s42003-022-04174-2 (PMC9640540; doi:10.1038/s42003-022-04174-2)
Supplement: Supplementary file 2 — Supplementary Information [file 42003_2022_4174_MOESM2_ESM.pdf]

SUPPLEMENTARY INFORMATION

An LH1–RC Photocomplex from an Extremophilic Phototroph Provides Insight into Origins of Two Photosynthesis Proteins

K. Tani, R. Kanno et al.

Supplementary Table 1 Comparison of the distances of His–BChl(Mg) and BChl(Mg)–BChl(Mg) in LH1, LH2 and RC special pairs from various phototrophic bacteria.

	Distance of His(Nε2) to BChl–Mg (Å)		Distance of Mg–Mg (Å)		
LH1 or LH2	α	β	Long	Short	PDB ID
Rpi. globiformis	**2.41**	**2.08**	**9.40**	**8.58**	**7XXF**
Rba. sphaeroides (Dimer)	2.58	2.21	9.45	8.36	7VY2
Rba. sphaeroides (Monomer)	2.58	2.20	9.55	8.37	7F0L
Rsp. rubrum (LH1)	2.27	2.03	9.34	8.51	7EQD
Rps. palustris (LH1-W)	2.93	2.71	9.61	8.2	6Z5S
Tch. tepidum (LH1)	2.19	2.19	8.88	8.72	5Y5S
Trv. strain 970 (LH1)	2.33	2.31	8.90	8.46	7C9R
Blc. viridis (LH1)	2.54	2.25	8.8	8.5	6ET5
Rfx. castenholzii (B880)	2.32	2.29	9.5	9.3	5YQ7
Rbl. acidophilus (B850)	2.34	2.34	9.5	8.8	1NKZ
Phs. molischianum (B850)	2.27	2.32	9.2	8.9	1LGH
RC (special pair)	L-subunit	M-subunit	BChl *a* (L)–BChl *a* (M)		
Rpi. globiformis	**2.25**	**2.24**	**7.73**		**7XXF**
Rba. sphaeroides (LH1–RC)	2.22	2.13	7.85		7VY2
Rba. sphaeroides (RC-only)	2.27	2.06	7.84		2J8C
Rsp. rubrum	2.09	2.12	7.76		7EQD
Rps. palustris	2.73	2.74	7.69		6Z5S
Tch. tepidum	2.17	2.19	7.87		5Y5S
Trv. strain 970	2.33	2.31	7.65		7C9R
Blc. viridis	2.36	2.35	7.83		6ET5

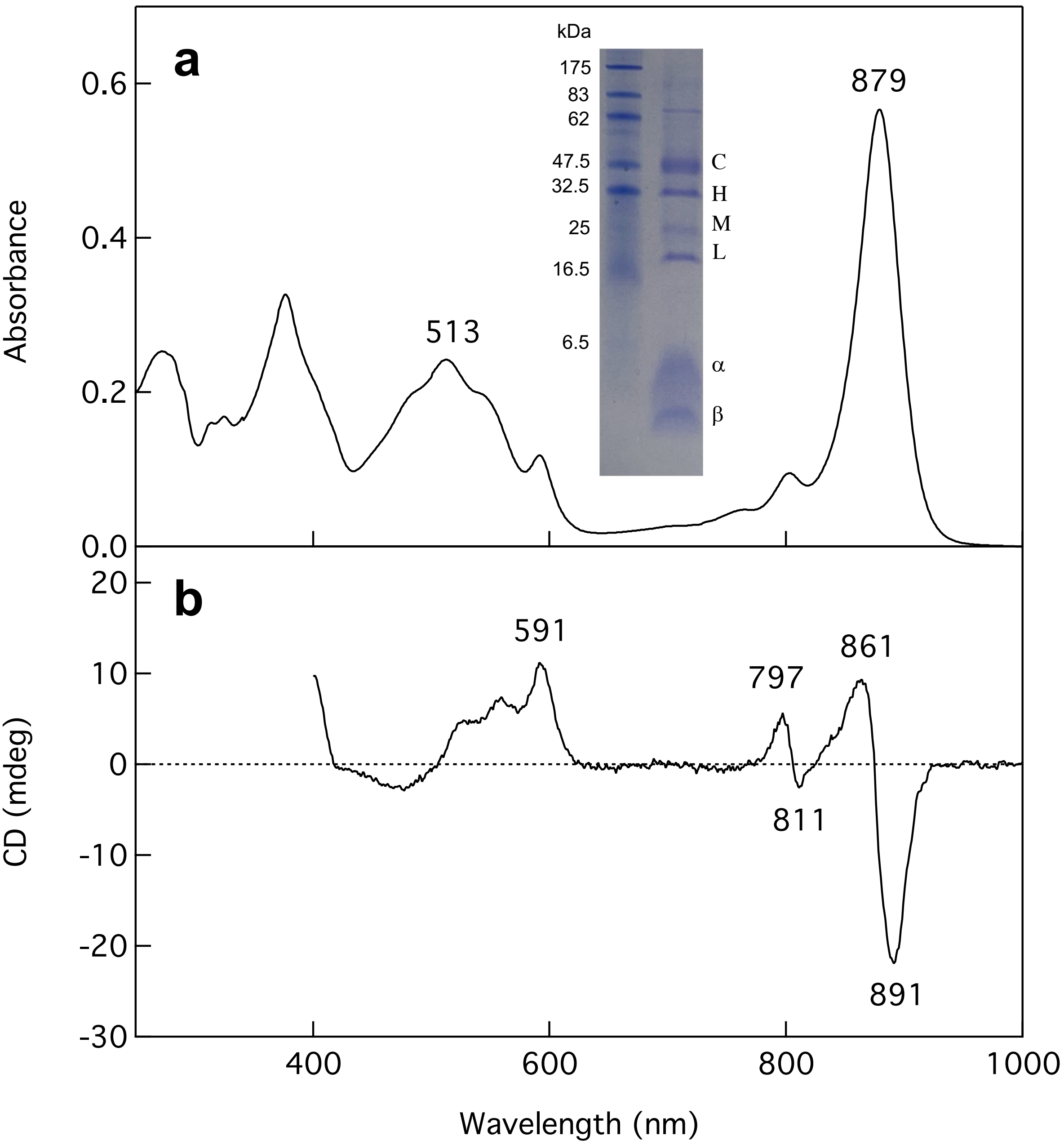

Supplementary Fig. 1 Room temperature absorption (a) and CD (b) spectra of the *Rpi. globiformis* LH1–RC complex. Inset: Coomassie blue-stained 12% SDS-PAGE gel for the purified LH1-RC with their assignments indicated. CD spectrum was recorded on a Jasco J-720w spectropolarimeter in the range from 400 nm to 1000 nm under the following conditions: 100 nm/min scan speed, 5 nm bandwidth, 1 sec response time, 5 scans.

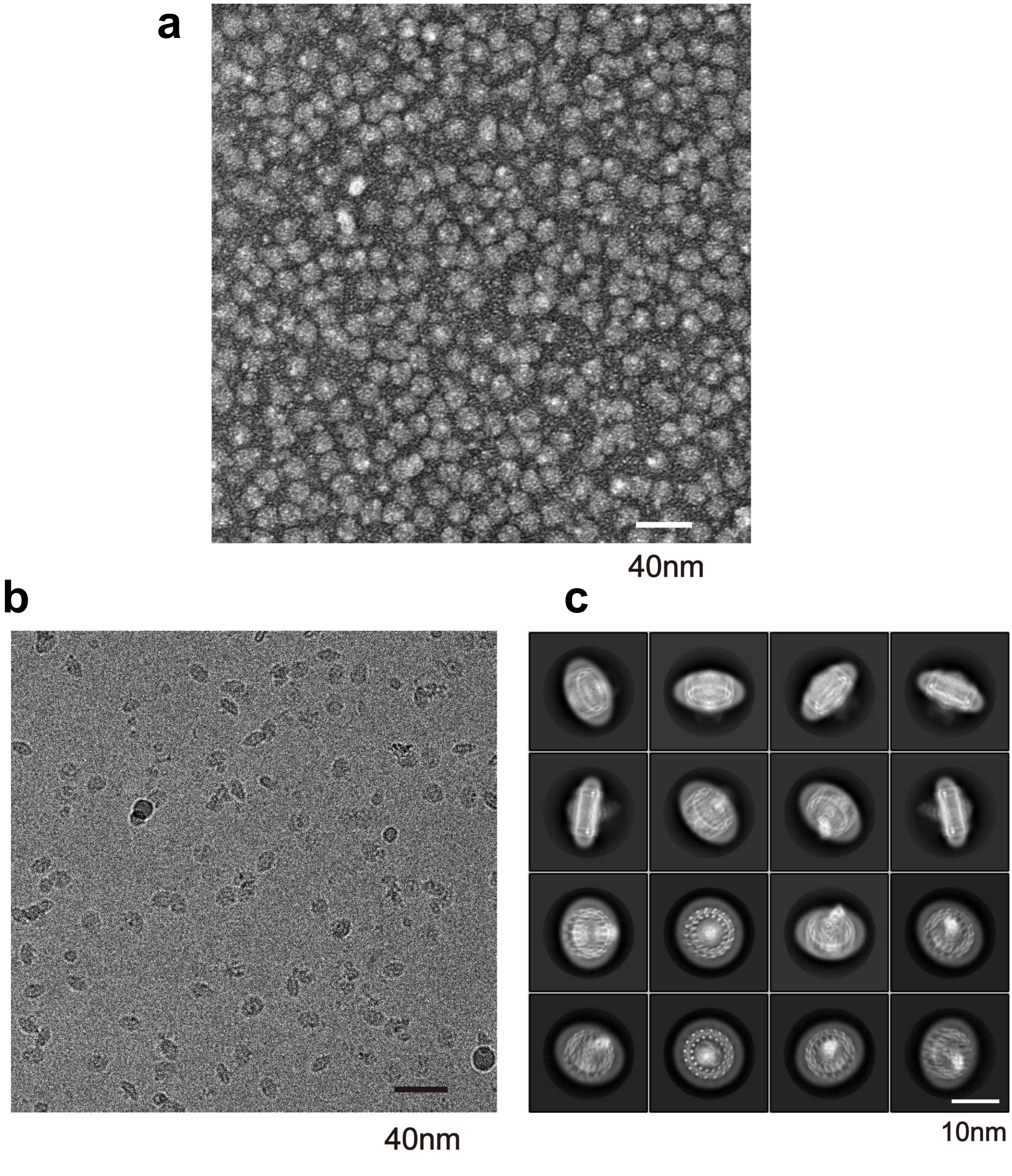

Supplementary Fig. 2 Electron micrographs of the *Rpi. globiformis* LH1–RC complex. A representative negatively stained **(a)** and cryo-EM **(b)** micrographs of the purified LH1–RC particles. **(c)** Representative 2D class averages from cryo-EM micrographs.

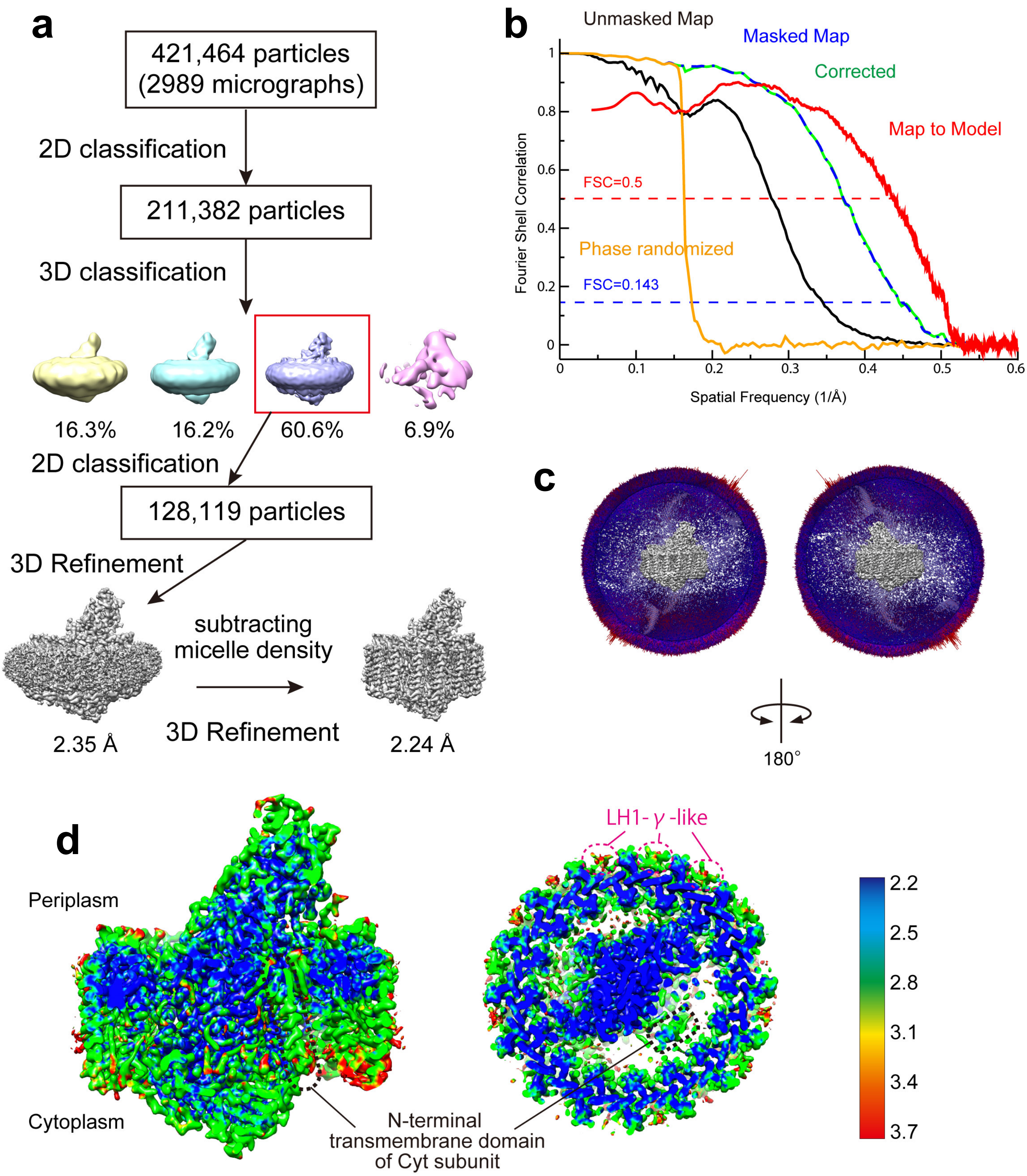

Supplementary Fig. 3 Structure determination of the *Rpi. globiformis* LH1–RC complex by cryo-EM. (a) Image processing flow of 3D classification and reconstruction. **(b)** The Fourier shell correlation (FSC) plots of the cryo-EM map (unmasked: black, masked: blue, phase randomized corrected: green, phase randomized: orange) and the FSC plot of the model versus the final map (red) are superimposed. **(c)** Angular distribution of reconstructed particles. **(d)** Local resolution representation of the LH1–RC structure. A longitudinal sectional view (*left*) and a central cross sectioned view (*right*) from periplasmic side. The map is shown in rainbow colors as shown in the right color bar. The contour level of the map raw density is 0.02.

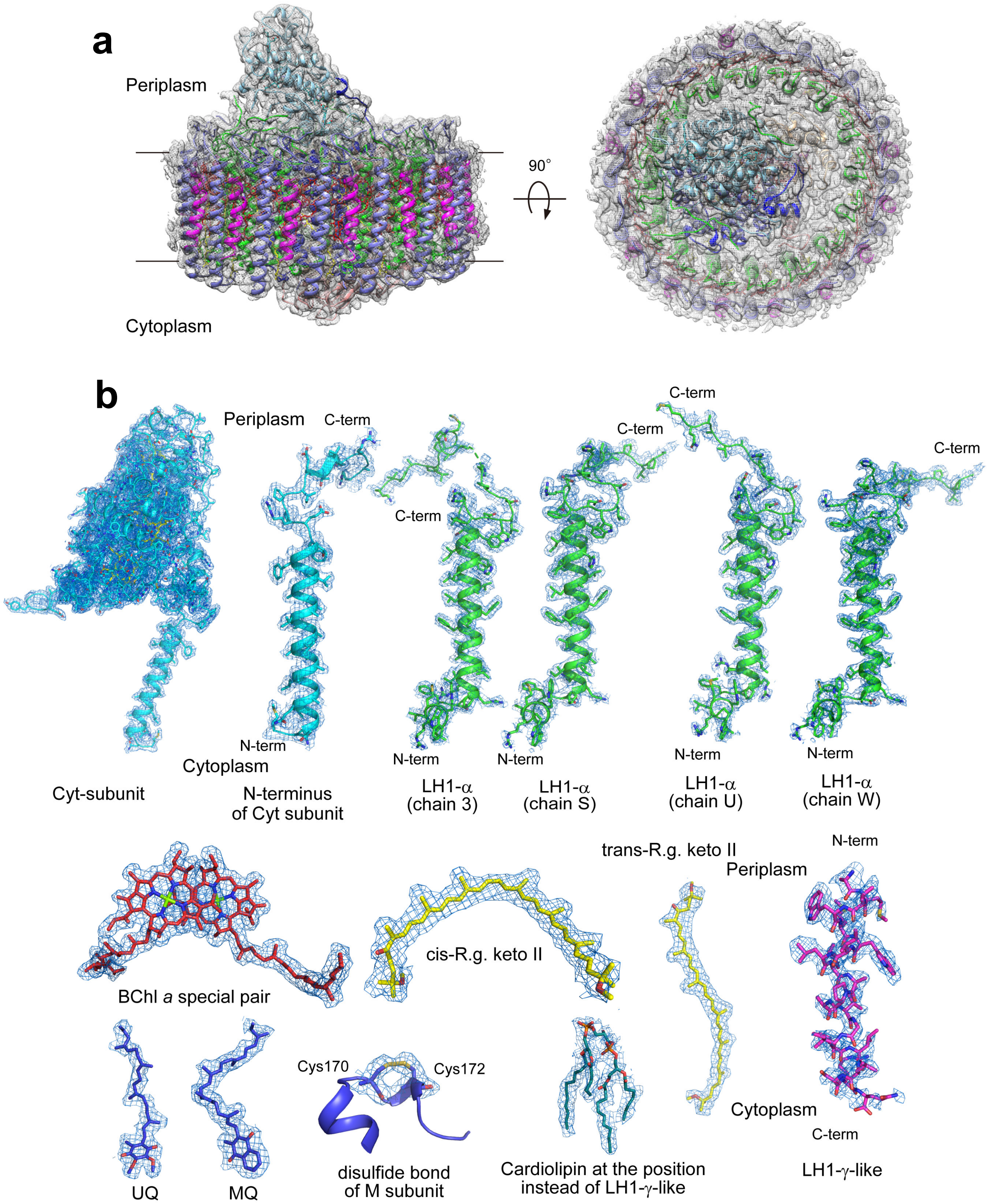

Supplementary Fig. 4 Cryo-EM densities and structural models of the *Rpi. globiformis* LH1–RC complex. **(a)** Overall structure of the LH1–RC complex. Cartoon representation of the complex with the cryo-EM density in gray mesh. Side view (*left*) parallel to the membrane plane and top view (*right*) from periplasmic side. **(b)** Selected polypeptides and cofactors. The density maps are shown at a contour level of 3.0 σ, except for the LH1 γ-like polypeptide and the disulfide bond of M subunit (4.0 σ). The color codes are the same as in Fig. 1.

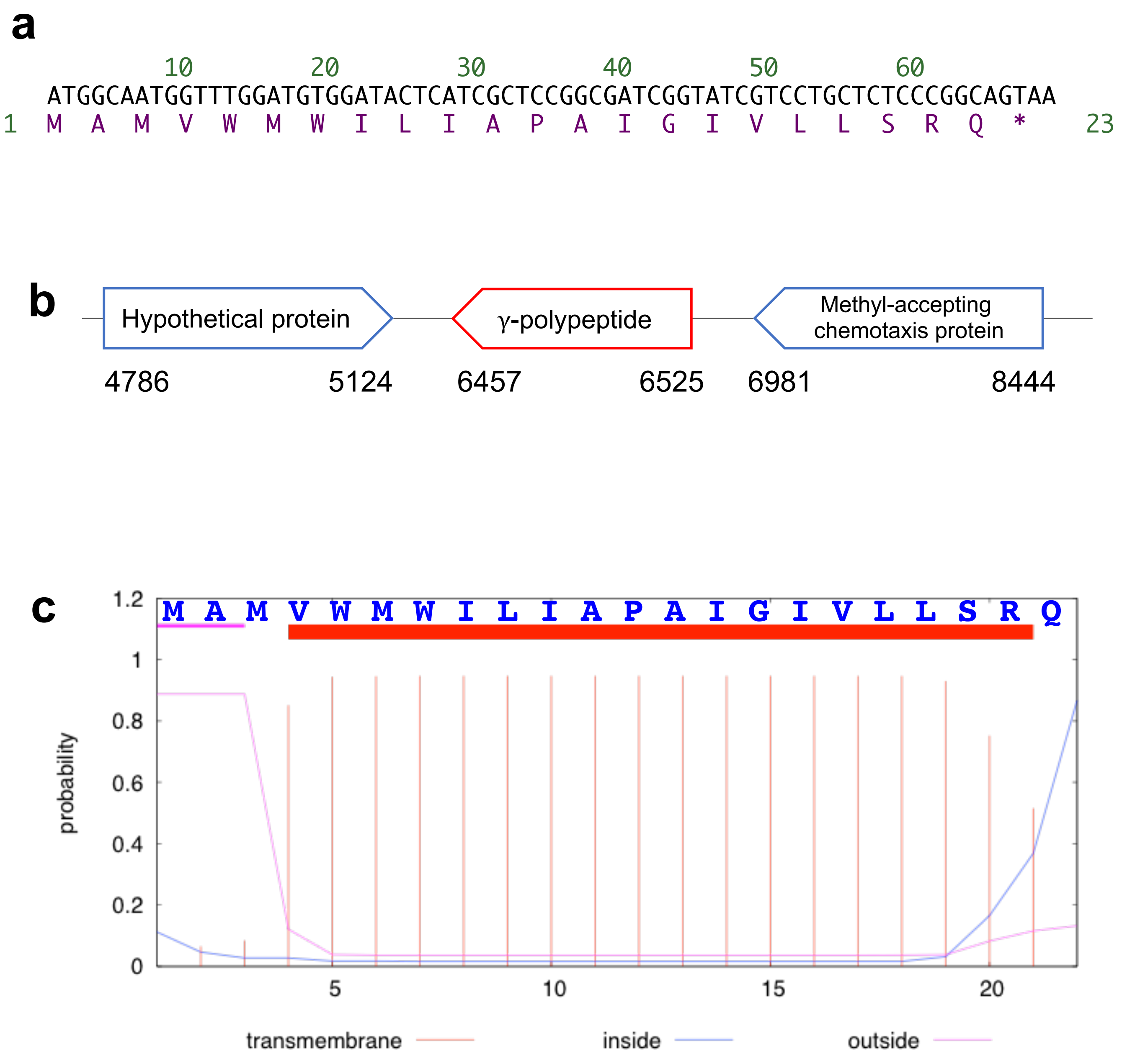

Supplementary Fig. 5 Genomic information and property of the *Rpi. globiformis* LH1 γ-like polypeptide. **(a)** DNA and translated amino acid sequences. **(b)** Arrangement of the gene encoding the γ-like polypeptide. **(c)** Transmembrane domain of the γ-like polypeptide predicted by the protein topology program TMHMM.

a

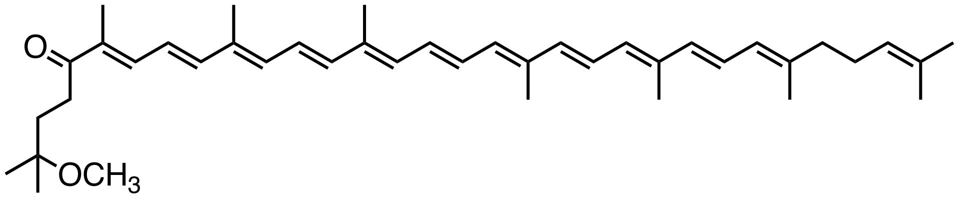

R.g.keto-I (1-methoxy-1,2-dihydro-ψ,ψ-caroten-4-one)

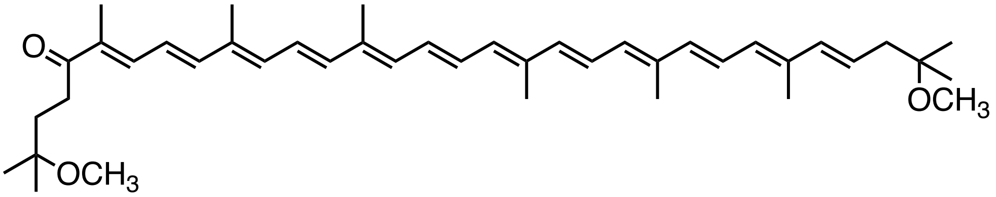

R.g.keto-II (1,1'-dimethoxy-3',4'-didehydro-1,2,1',2'-tetrahydro-ψ,ψ-caroten-4-one)

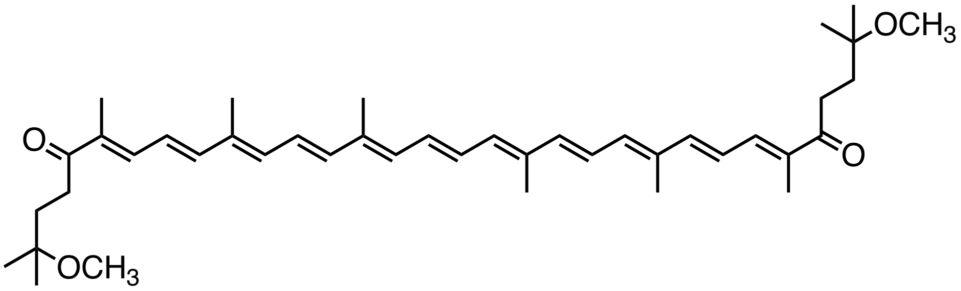

R.g.keto-III (1,1'-dimethoxy-1,2,1',2'-tetrahydro-ψ,ψ-carotene-4,4'-dione)

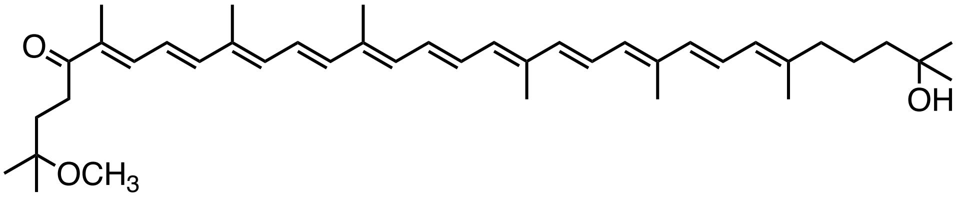

R.g.keto-IV (1'-hydroxy-1-methoxy-1,2,1',2'-tetrahydro-ψ,ψ-caroten-4-one)

b

Carotenoid	Membrane	**LH1–RC**	LH2
R.g. keto-I	2	3	2
**R.g. keto-II**	71	**84**	61
R.g. keto-III	14	4	21
R.g. keto-IV	12	9	17

Supplementary Fig. 6 Keto-carotenoids in *Rpi. globiformis*. **(a)** Molecular structures. **(b)** Carotenoid compositions (mol % of total carotenoids) determined in this work for the membrane, purified LH1–RC and LH2 complexes.

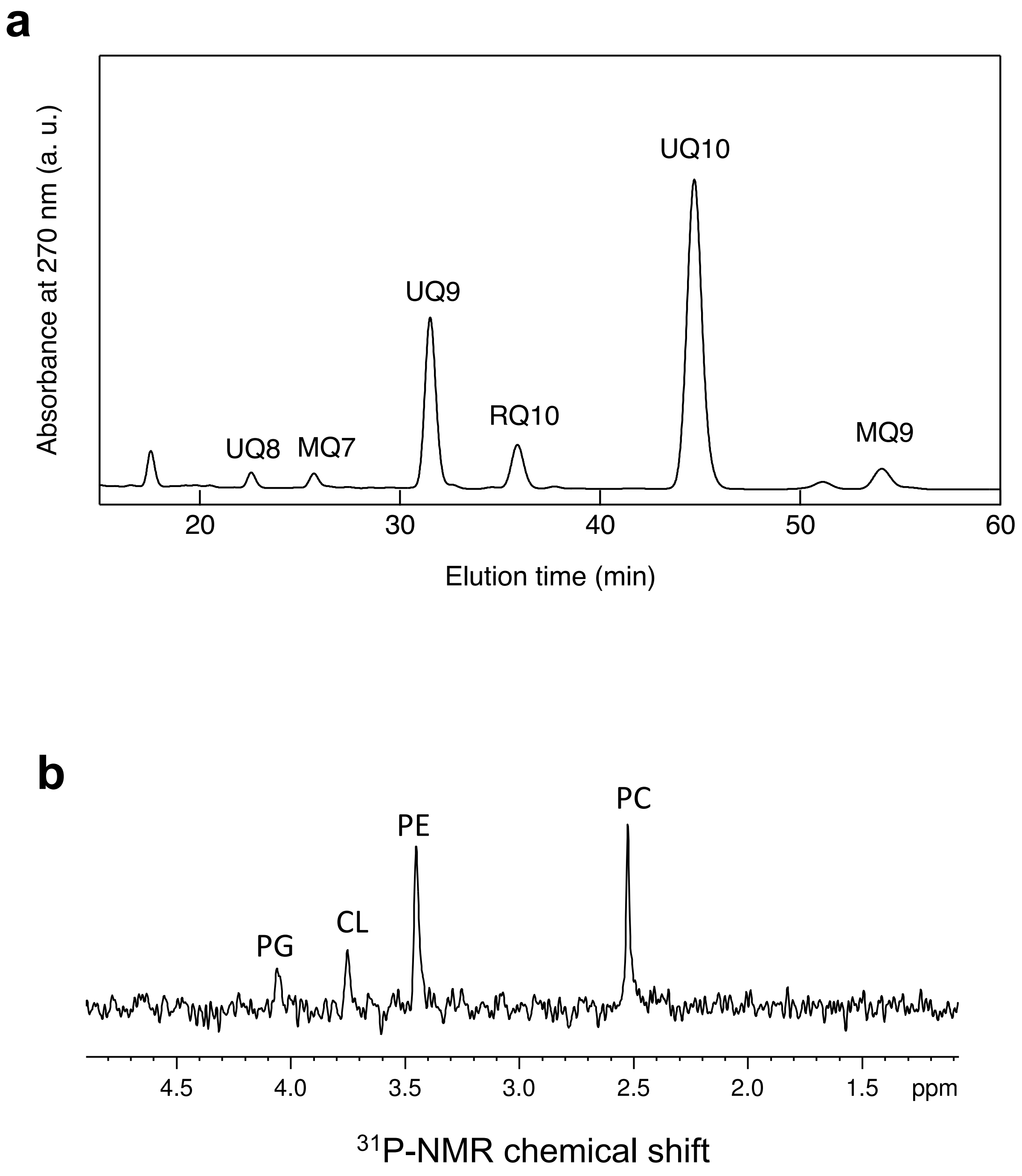

Supplementary Fig. 7 Characterizations of the quinones and phospholipids in *Rpi. globiformis*. (**a**) Reverse-phase HPLC chromatogram (TOSOH, TSKgel ODS-80Ts, 4.6 × 250 mm) of the quinones extracted from chromatophores isocratically eluted at 25 ˚C by 7:3 methanol/isopropanol at flow rate of 0.7 mL/min. (**b**) ^{31}P-NMR spectrum of the phospholipids extracted from chromatophores. PG: phosphatidylglycerol, CL: cardiolipin, PE: phosphatidylethanolamine, PC: phosphatidylcholine.

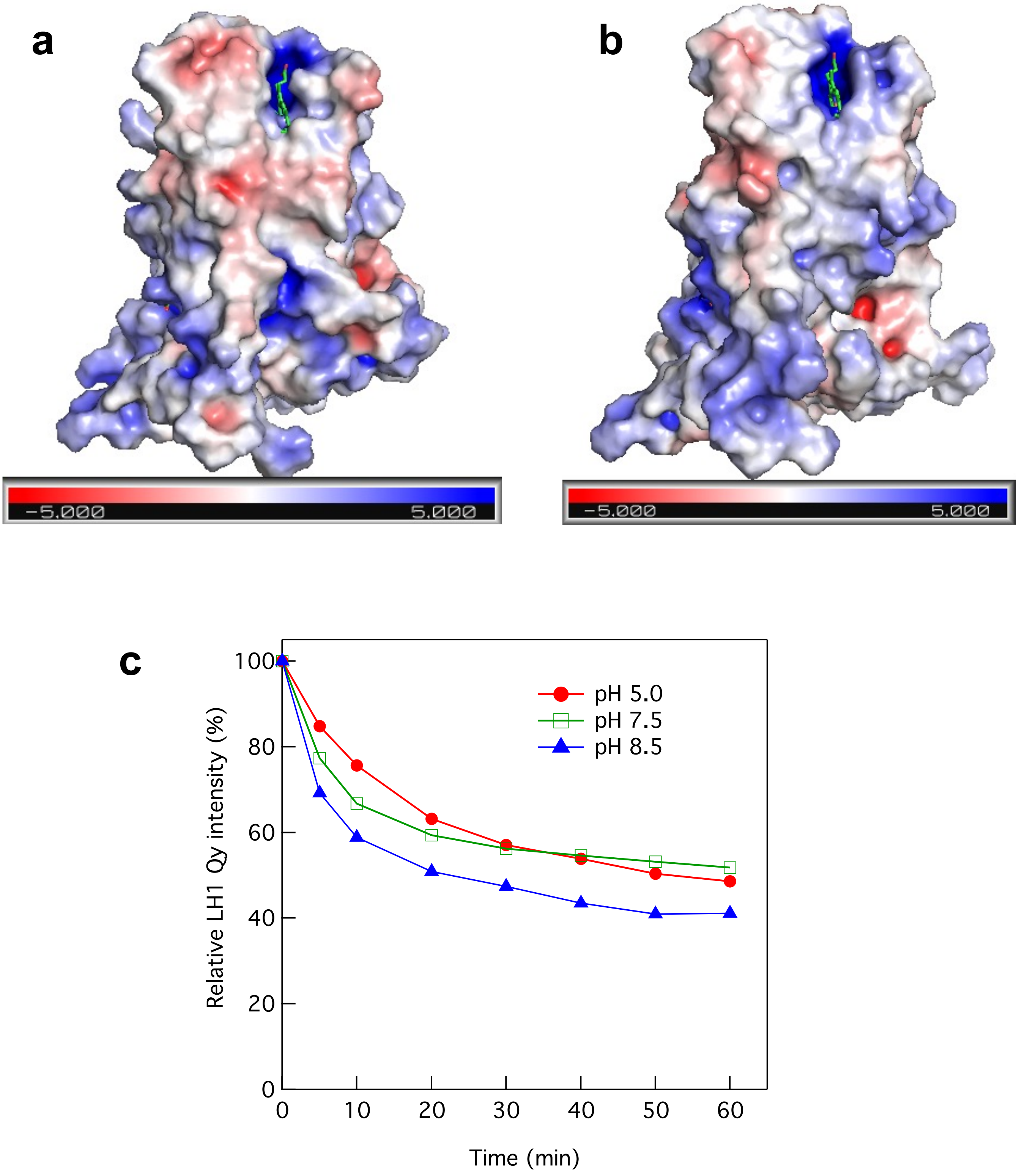

Supplementary Fig. 8 Surface charge distributions of the Cyt subunits from other bacteria and thermostability of the *Rpi. globiformis* LH1–RC complex. **(a)** Surface charge distribution (from –5.0 k_BT to +5.0 k_BT) of the Cyt subunit in *Blc. viridis* RC (PDB: 3T6E). **(b)** Surface charge distribution (from -5.0 k_BT to +5.0 k_BT) of the Cyt subunit in *Tch. tepidum* RC (PDB: 5Y5S). **(c)** pH-dependence of the thermostability of the *Rpi. globiformis* LH1–RC measured at 70 ˚C in 20 mM Tris-HCl containing 0.05% DDM and 200 mM NaCl.

a

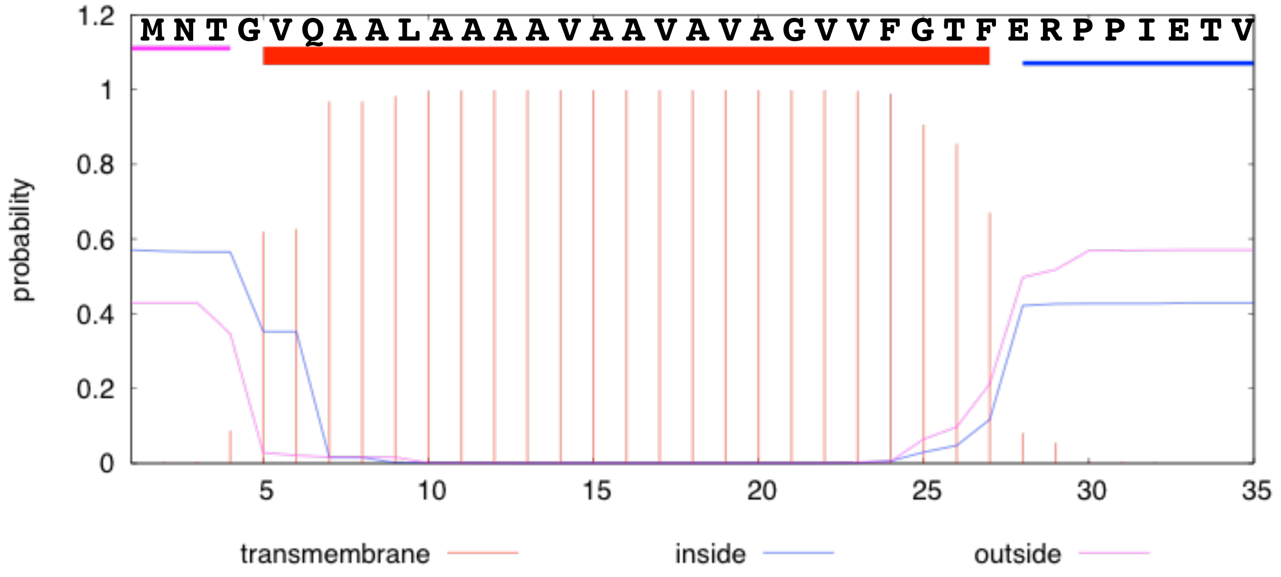

b

```
Rba_sphaeroides PufX    HLNTNPKTNLRLWVAFQMMKGAGWAGGVFFGTLLLIGFFRVVGRMLPIDENPAPAPNITG
Rpi_globiformis Cyt     -MNTG----VQAALAAAAVAAVAVAG-VVFGTFERP----------PIETVQRGA-----
                         :**.    ::  :*   : ... ** *.***:             **:     *
```

Supplementary Fig. 9 Property of the N-terminal domain of the *Rpi. globiformis* Cyt subunit and sequence comparison with the *Rba. sphaeroide*s (IL106) PufX. (a) Prediction of transmembrane domain of the intact N-terminus of the *Rpi. globiformis* Cyt subunit by protein topology program TMHMM. **(b)** Alignment of the amino acid sequences around the transmembrane domains between *Rba. sphaeroide*s PufX and *Rpi. globiformis* Cyt subunit using ClustalW. Identical amino acids (*), conservative (:) and semi-conservative (.). Transmembrane regions are underlined.

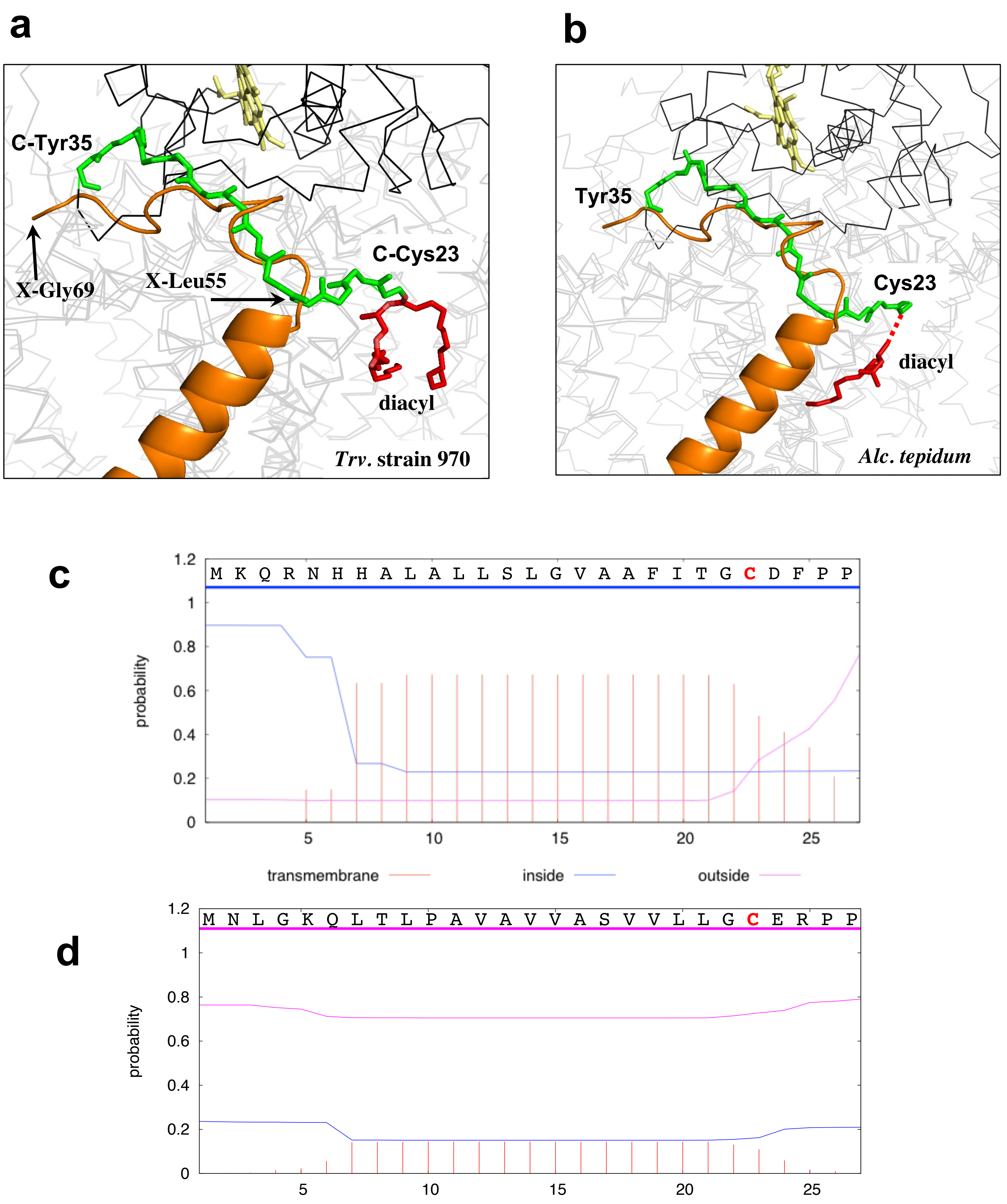

Supplementary Fig. 10 Structural similarities between PufX and the truncated N-termini of the *Alc. tepidum* and *Trv*. strain 970 Cyt subunits. Superpositions of the Cα carbons of the RC-M subunit of the *Rba. sphaeroides* (PDB: 7F0L) with those of *Alc. tepidum* (**a**, PDB: 7VRJ) and *Trv*. strain 970 (**b**. PDB: 7C9R). The *Alc. tepidum* and *Trv*. strain 970 Cyt subunits are represented by black ribbon with their N-terminal regions (main chain from Cys23 to Tyr35) shown by green sticks, the covalently bound diacyl groups by red sticks. Other portions of the two complexes are shown by gray ribbons. Hydrophobicity analyses for the truncated N-termini of Cyt subunits are show in **(c)** for *Alc. tepidum* and **(d)** for *Trv*. strain 970 with their sequences on the top by TMHMM. The Cys residues that bind a diacyl group are shown in red font.

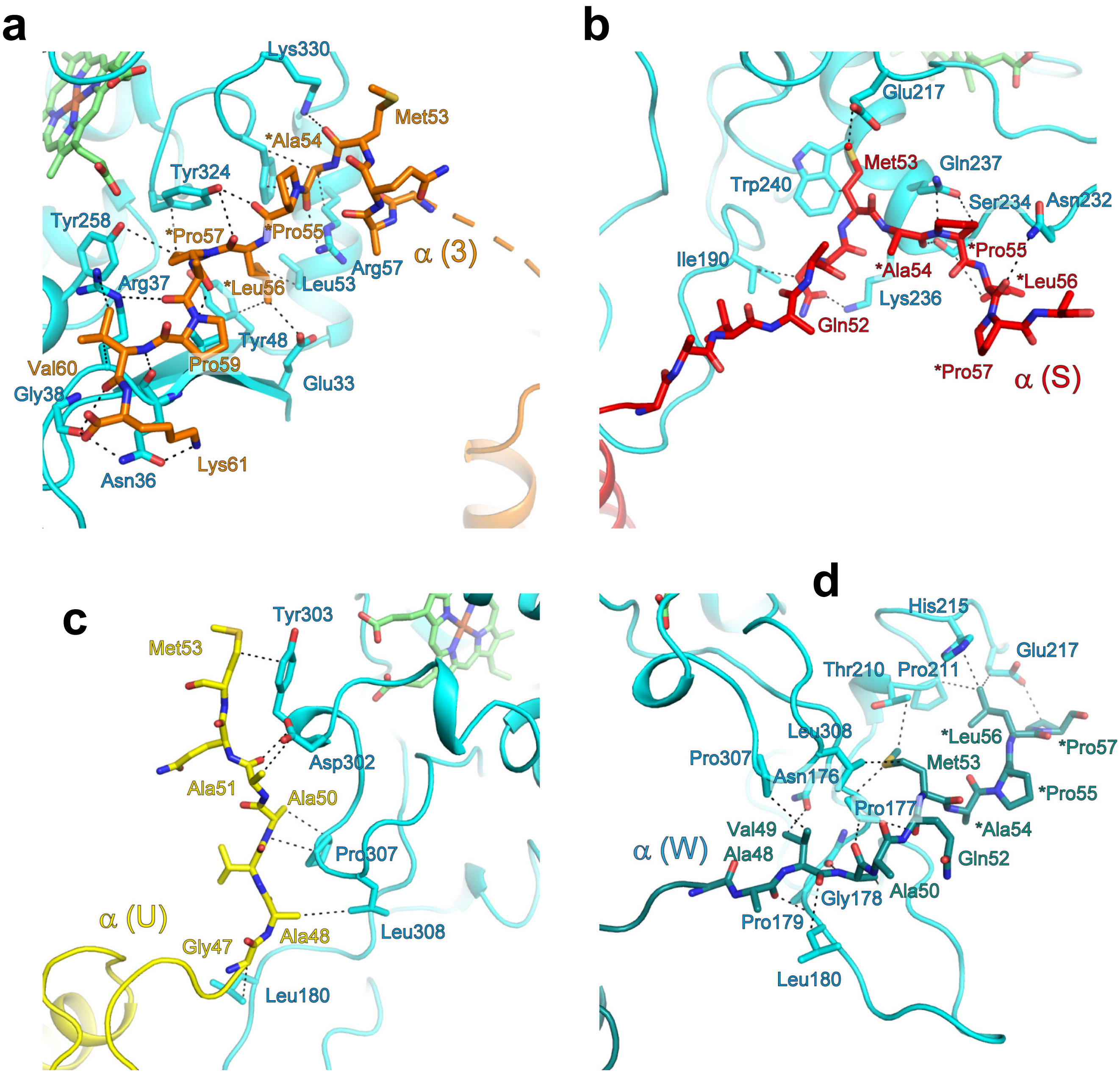

e

```
  1 MNTGVQAALAAAAVAAVAVAGVVFGTFERPPIETVQRGARGLAMSELYNPRFLAETRAEN  60
 61 VVPASLPRLPDVGLKAGEVYHNVQVLKDVSVGNFTRLMASMTTWVAPQQGCGYCHNTNNM 120
121 ASDAKYTKVVARRMIQMVQHINQDWKVHVMANAPTGVVCYTCHRGNPVPKNIWFNNPGPL 180
181 QAGGYAEAEIGKNHPAPFANNSSLPLDPFTPFLEHAENIRVQATQALPGTDNSSIKQTYW 240
241 TYALMASFTQALGVNCTYCHDSRLWESWDMAPPQRVTAWYGIRMVRDLNNNFLDPLKTTF 300
301 PDYRRGPLGDSPKVWCATCHNGVYKPLFGKSMVTTFPELTKVSQ
```

Supplementary Fig. 11 Interactions between the *Rpi. globiformis* Cyt subunit and four LH1 α-polypeptides. **(a)** with chain 3 (orange), **(b)** with chain S (red), **(c)** with chain U (yellow), **(d)** with chain W (turquoise blue). Cyt subunit, cyan; Heme, yellow-green sticks. Dashed lines represent typical close contacts (< 4.0 Å) between the proteins. The amino acid residue in the key motif [A/S]-[P/A]-[L/M/I]-P is marked with an asterisk (*). **(e)** Amino acid sequence of the *Rpi. globiformis* Cyt subunit with the heme-binding motifs marked by red fonts.

a

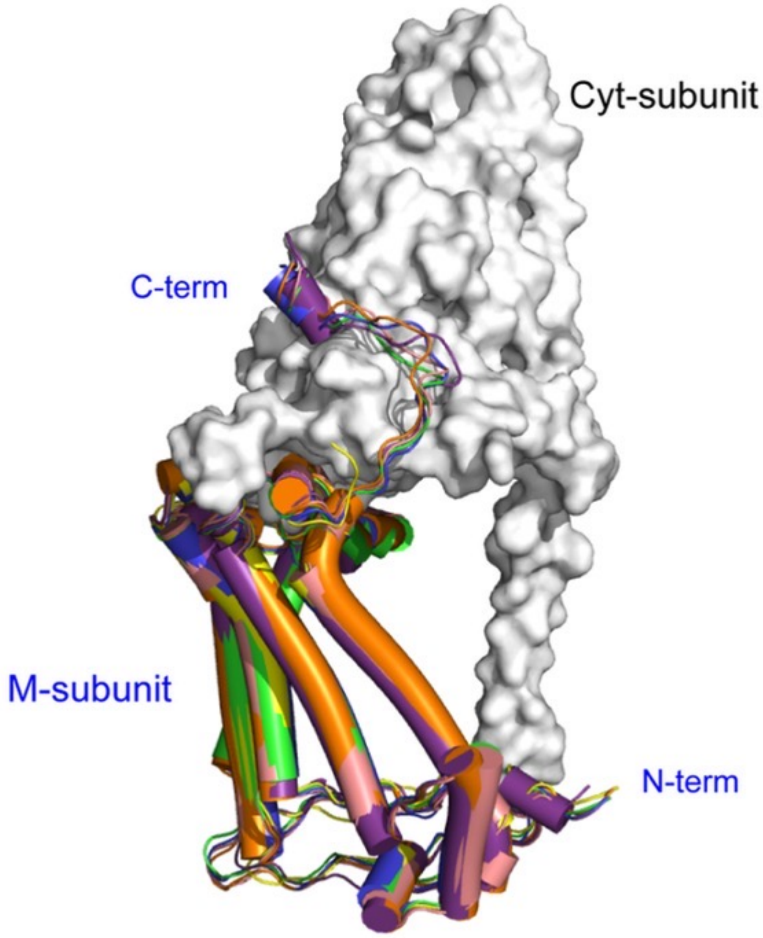

b

```
Rpi. globiformis   292 DNWFLWGVKHGIAAPWPNVFPHVVDPALLATGVGK 326
Blc. viridis       290 DNWYLWCVKHGAAPDYPAYLPATPDPASLP-GAPK 323
Trv. strain 970    292 DNWYLWAVKHGVAPSYPSELT-IDNPYLTQG-VAQ 324
Tch. tepidum       292 DNWYLWAVKHGMAPAYPEVVT-AVNPYETAAEVMQ 325
Alc. tepidum       291 ENWYLWAIKHGVAPAYPEVVT-AVDPYATATGVTQ 324
                       :**:** :*** *. :*  ..   :*      . :

Rba. sphaeroides   293 DNWYVWGQNHGMAPLN------------------- 308
```

Supplementary Fig. 12 Sequence information of the C-terminus of M-subunit. (a) Superposition of the M-subunit of *Rpi. globiformis* (blue), *Blc. viridis* (PDB: 6ET5, purple), *Trv.* strain 970 (PDB: 7C9R, orange), *Tch. tepidum* (PDB: 5Y5S, green), *Alc. tepidum* (PDB: 7VRJ, salmon) and *Rba. sphaeroides* (PDB: 7F0L, yellow). Each M-subunit is shown in cartoon, and the Cyt subunit of *Rpi. globiformis* is shown in surface representation. **(b)** Sequence comparison of the C-terminus of M subunit between purple sulfur bacteria *Rpi. globiformis*, *Blc. viridis, Trv.* strain 970, *Tch. tepidum, Alc. tepidum* and *Rba. sphaeroides*. A red dashed rectangle indicates unique region for the Cyt subunit bound RC. Blue rectangle and line indicate helix and coil region, respectively. Symbol scheme: (*) identical, (:) highly similar, (.) similar.

a

LH1-α
formyl-MWRMWLLFDPRRILVALGVFLFVLALLIHFILLSTDRFNWLDGPHRGAVAAQMAPLPAPVK

LH1-β
MTPGGPSITGLTEAEAKEFHGIFITSFIVFTVIAIVAHLLAWQWRPWLPAVTGYGTAMNDAVSFIHATISQLA

LH1 γ-like polypeptide
formyl-MAMVWMWILIAPAIGIVLLSRQ

b

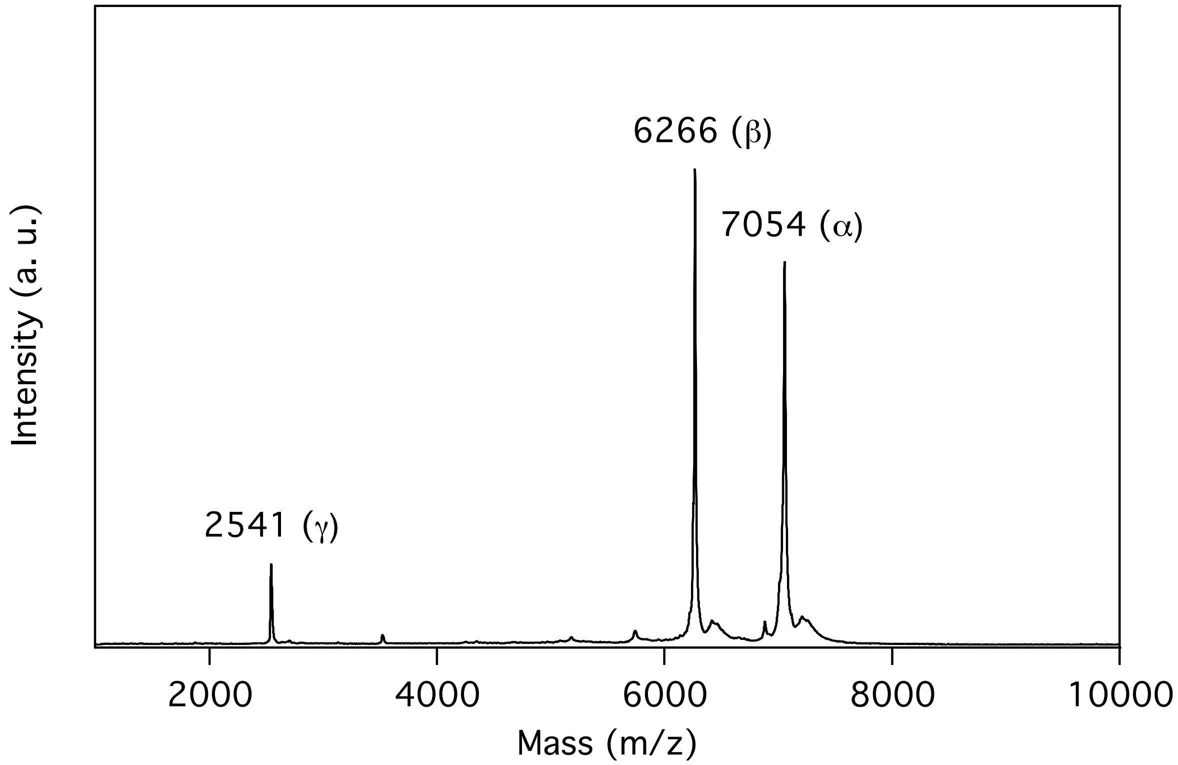

Supplementary Fig. 13 Characterizations of the *Rpi. globiformis* LH1 polypeptides. **(a)** Sequences of the LH1 polypeptides. Residues with gray fonts were not identified in the purified proteins. **(b)** MALDI/TOF-MS spectrum of the purified LH1–RC.

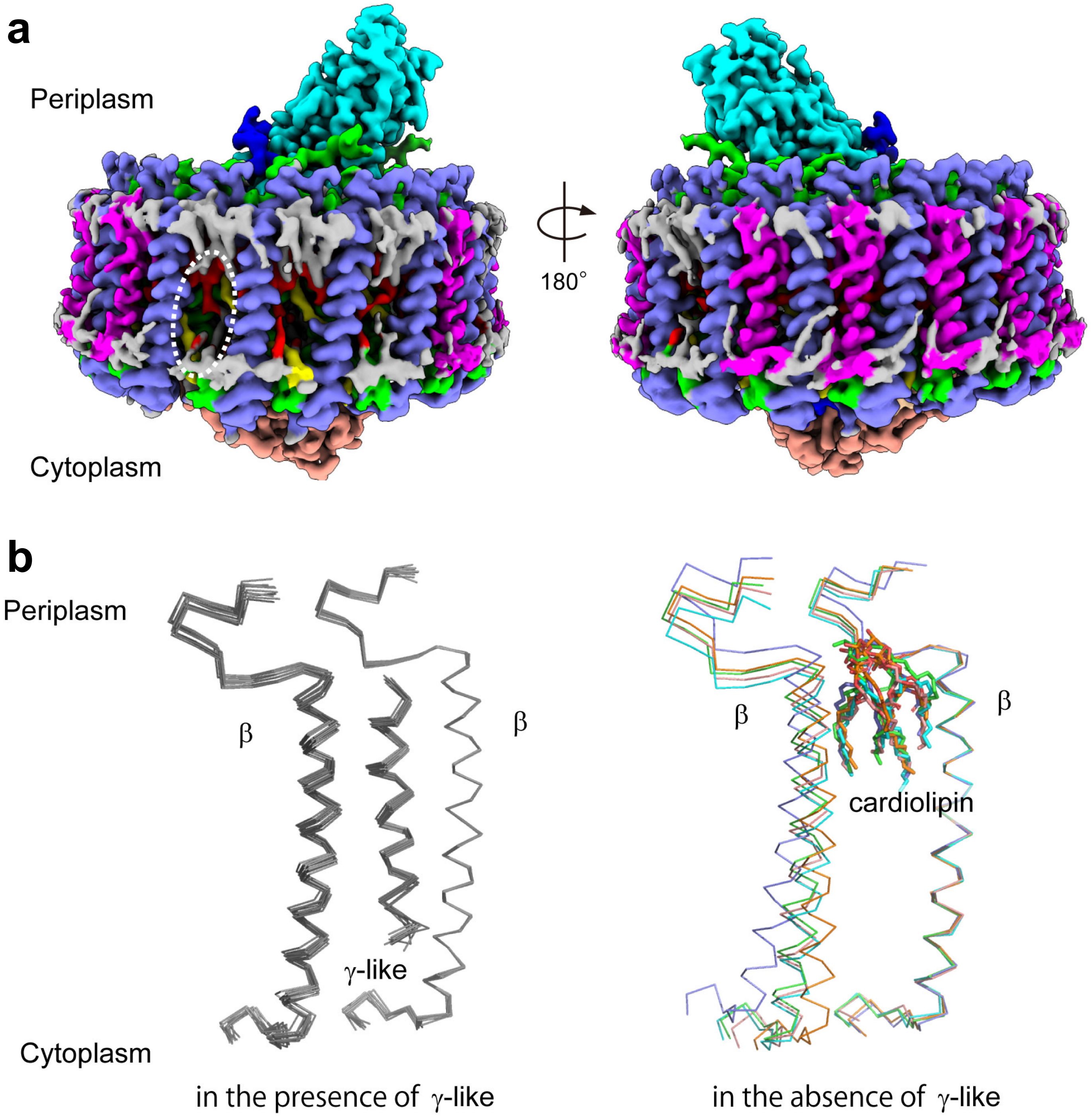

Supplementary Fig. 14 Structural characterization of the LH1 interfaces for the *Rpi. globiformis* LH1–RC complex. (a) Overall structure of the LH1–RC complex. Side views of surface representation of the complex with the color code as in Fig. 1. A dotted oval indicates an interface lacking the γ-like polypeptide. **(b)** Structural comparison of LH1 β–β interfaces by superimposing the Cα atoms of LH1-β transmembrane region. The interfaces of LH1-βs that bind γ-like polypeptides are converged (dark gray). By contrast, the interfaces of LH1-βs in the absence of γ-like polypeptides are significantly diverged (green, cyan, salmon, orange or slate), but the cardiolipins (shown as sticks) sandwiched between the LH1-βs are located at the similar position and adopt similar conformation.

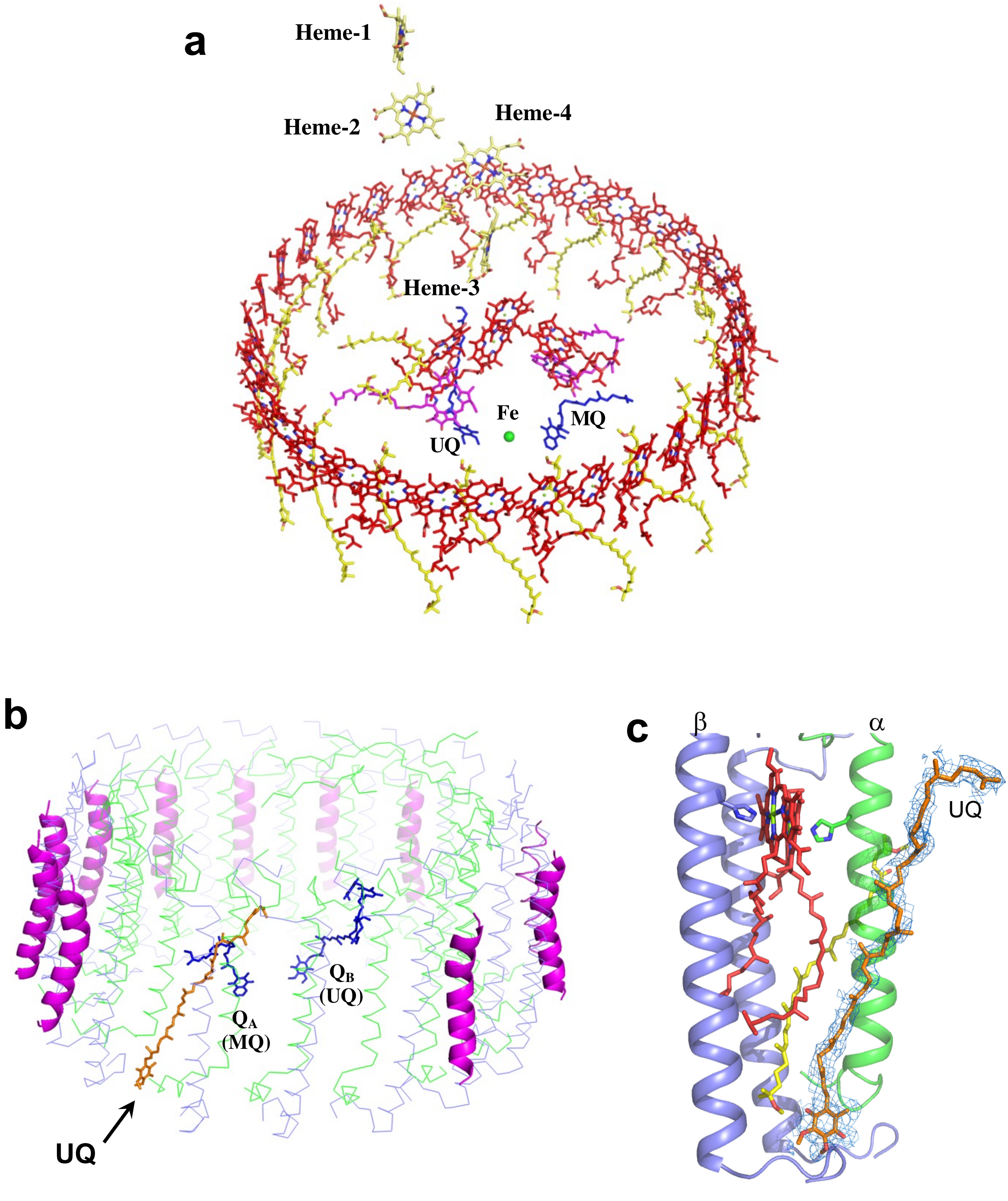

Supplementary Fig. 15 Arrangement of the co-factors in the *Rpi. globiformis* LH1–RC. (a) Tilted view of the co-factors. BChl *a*, red; carotenoids, yellow; BPhe *a*, magenta; quinones, blue. **(b)** A UQ molecule inserted in the channel formed by two LH1-αβ pairs where lacks a γ-like polypeptide. **(c)** Density map of the additional UQ molecule with the neighboring LH1 subunits. The density maps are shown at a contour level of 3.0 σ.

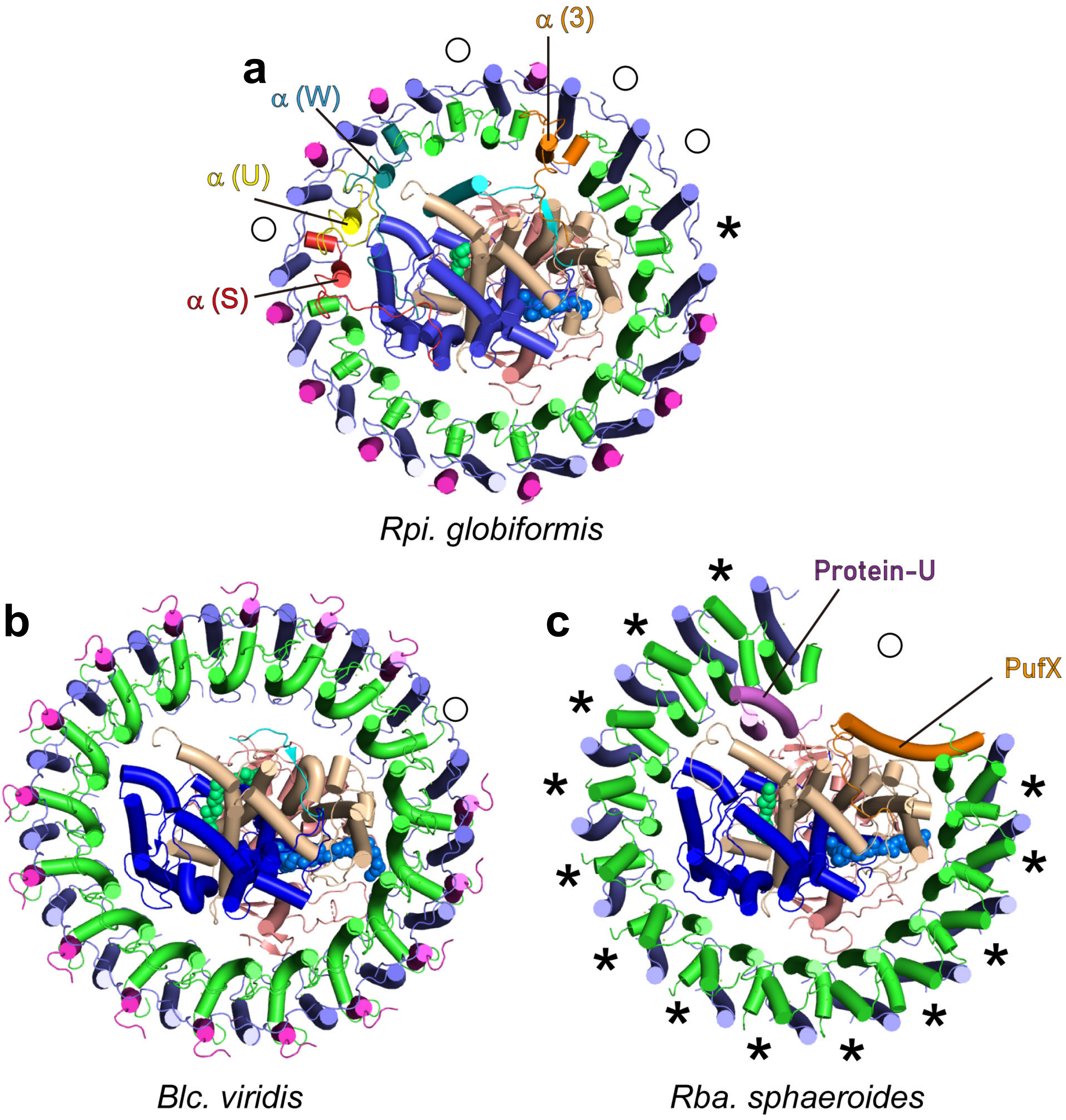

Supplementary Fig. 16 Putative quinone channels in the LH1–RC complexes. Top views from the periplasmic side of LH1–RCs from *Rpi. globiformis* **(a)**, *Blc. viridis* (PDB: 6ET5) **(b)** and *Rba. sphaeroides* (PDB: 7F0L) **(c)**. Open circles or asterisks indicate open or blocked channels with cofactors, respectively. The asterisk in **(a)** indicates the channel that is blocked by a carotenoid and a UQ molecule as shown in Supplementary Fig. 15 (b, c). LH1-α, green (In *Rpi. globiformis,* chain 3, orange; chain S, red; chain U, yellow; chain W, turquoise blue); LH1-β, slate; LH1-γ or LH1 γ-like polypeptide, magenta; L-subunit, wheat; M-subunit, blue; H-subunit, salmon; Cyt-subunit, cyan. For clarity, the N-terminus of Cyt-subunit is only shown. Quinones at Q_A and Q_B site are shown in marine and lime-green sphere models, respectively. In *Rba. sphaeroides*, PufX, orange; Protein-U, violet.

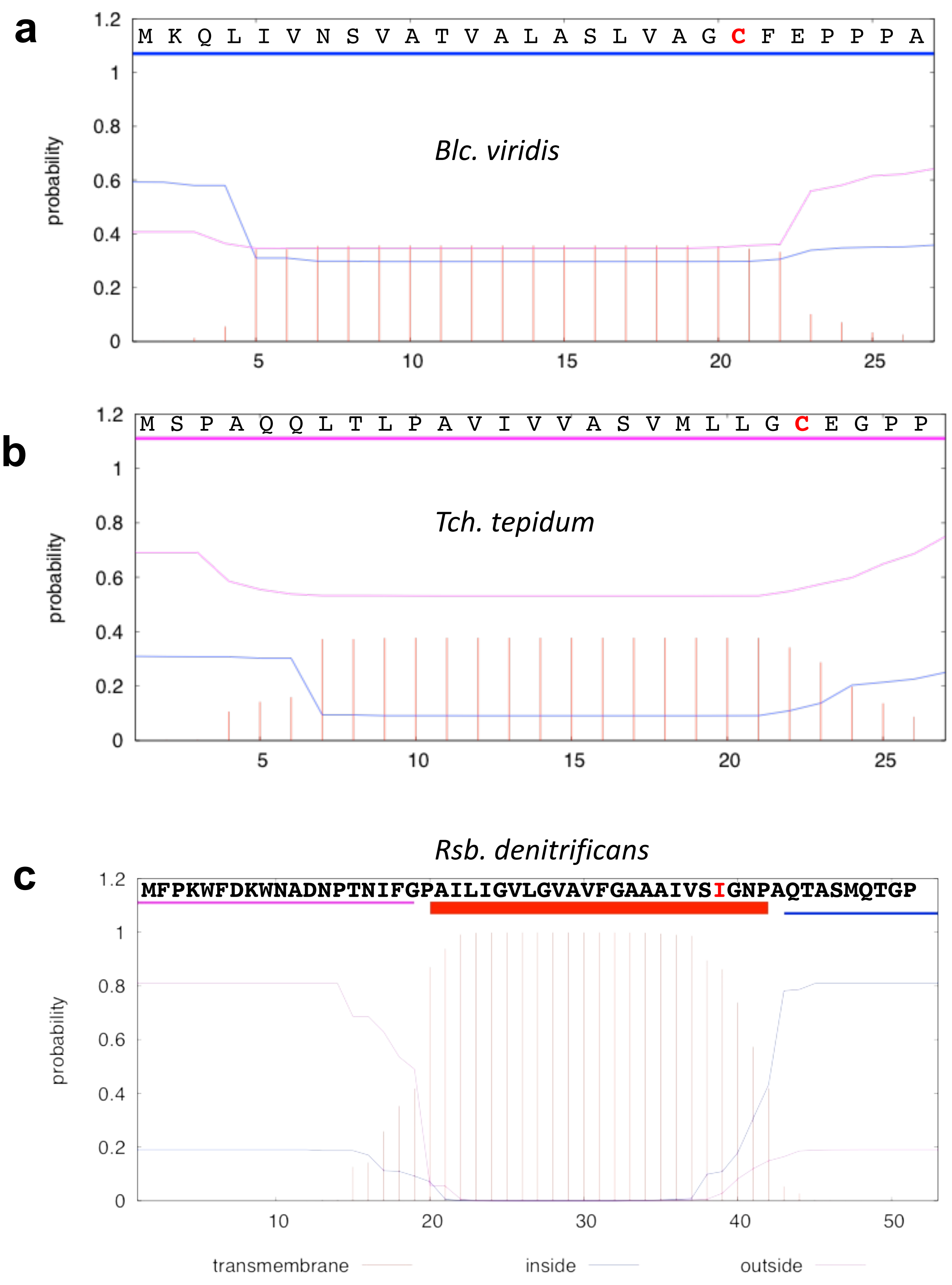

Supplementary Fig. 17 Predictions of transmembrane domains for the truncated and intact N-termini of Cyt subunits in purple bacteria by TMHMM. (a) Truncated fragment for the *Blc. viridis* with the sequence on the top. The Cys residue (red font) indicates truncation site. **(b)** Truncated fragment for *Tch. tepidum*. **(c)** A transmembrane domain is predicted for the *Rsb. denitrificans* Cyt subunit that has an Ile (red font) at the corresponding position of the Cys.
